# Impact of Machine Perfusion on the Immune Response After Liver Transplantation – A Primary Treatment or Just a Delivery Tool

**DOI:** 10.3389/fimmu.2022.855263

**Published:** 2022-07-08

**Authors:** Rebecca Panconesi, Mauricio Flores Carvalho, Daniele Dondossola, Paolo Muiesan, Philipp Dutkowski, Andrea Schlegel

**Affiliations:** ^1^ Department of Clinical and Experimental Medicine, Hepatobiliary Unit, Careggi University Hospital, University of Florence, Florence, Italy; ^2^ General Surgery 2U-Liver Transplant Unit, Department of Surgery, A.O.U. Città della Salute e della, Scienza di Torino, University of Turin, Turin, Italy; ^3^ General and Liver Transplant Surgery Unit, Fondazione IRCCS Ca’ Granda, Ospedale Maggiore, Policlinico and University of Milan, Milan, Italy; ^4^ Department of Surgery and Transplantation, Swiss Hepato-Pancreato-Biliary (HPB) Center, University Hospital Zurich, Zurich, Switzerland

**Keywords:** ischemia reperfusion injury, innate immune activation, machine perfusion, mitochondrial injury, hypothermic oxygenated perfusion, marginal livers

## Abstract

The frequent use of marginal livers forces transplant centres to explore novel technologies to improve organ quality and outcomes after implantation. Organ perfusion techniques are therefore frequently discussed with an ever-increasing number of experimental and clinical studies. Two main approaches, hypothermic and normothermic perfusion, are the leading strategies to be introduced in clinical practice in many western countries today. Despite this success, the number of studies, which provide robust data on the underlying mechanisms of protection conveyed through this technology remains scarce, particularly in context of different stages of ischemia-reperfusion-injury (IRI). Prior to a successful clinical implementation of machine perfusion, the concept of IRI and potential key molecules, which should be addressed to reduce IRI-associated inflammation, requires a better exploration. During ischemia, Krebs cycle metabolites, including succinate play a crucial role with their direct impact on the production of reactive oxygen species (ROS) at mitochondrial complex I upon reperfusion. Such features are even more pronounced under normothermic conditions and lead to even higher levels of downstream inflammation. The direct consequence appears with an activation of the innate immune system. The number of articles, which focus on the impact of machine perfusion with and without the use of specific perfusate additives to modulate the inflammatory cascade after transplantation is very small. This review describes first, the subcellular processes found in mitochondria, which instigate the IRI cascade together with proinflammatory downstream effects and their link to the innate immune system. Next, the impact of currently established machine perfusion strategies is described with a focus on protective mechanisms known for the different perfusion approaches. Finally, the role of such dynamic preservation techniques to deliver specific agents, which appear currently of interest to modulate this posttransplant inflammation, is discussed together with future aspects in this field.

## Introduction

The introduction of an effective immunosuppression was a milestone in the history of organ transplantation. Various modifications of the pharmaceuticals used to modulate the immune system have been reported for most solid organs since that. The general drawback remains however with the putative risk to develop de-novo malignancies, chronic renal failure, and cardiovascular or metabolic diseases (1, 2). In an attempt to reduce such side effects, the weaning of immunosuppression (IS) and the induction of graft tolerance are important targets nowadays (1, 3, 4). Human liver allografts display several unique immunological features, including transplantation across a positive cross match, less vigorous immunosuppressive regimens, the lack of a significant benefit from HLA matching, and the overall low rates of chronic rejection. While some of these characteristics can be explained by the unique regenerative capacity of the liver, only in liver transplantation a significant proportion of patients can be eventually discontinued from maintenance IS without immediate rejection, a phenomenon known as operational tolerance (5). Tailored posttransplant protocols will include biopsies and IS regimens, which are required to individualize such concepts for a successful IS minimization (6). This approach starts however with preventive measures, applied ideally before graft reperfusion to reduce the initial “inflammatory hit” during ischemia-reperfusion. This early activation of the innate immune system is the direct consequence of ischemia-reperfusion-injury (IRI), which is the initial trigger of tissue inflammation in the transplanted organ and also in the entire recipient (7). This complex interplay between graft and recipient triggers various less well explored crosstalks between implanted organ and recipient’s immune system and promotes the establishment of an inflammatory milieu with a chronic component (8, 9). The level of this inflammation strictly depends on the initial donor and organ quality and can be modulated through a different preservation strategy (10). The potential role of machine perfusion technology and the effect on this initial posttransplant inflammation, which is crucial for recipient outcomes, is therefore highly relevant (9). While the first randomized controlled trials (RCT) are now available and demonstrate a reduction of posttransplant complications with the use of machine perfusion, the impact on basic mechanisms of early IRI and subsequent immune response, remains not well explored.

This review focus therefore on the mechanisms of how the innate immune response is triggered after liver transplantation and the role of machine perfusion techniques, individual and as a tool to deliver therapeutic agents to the organ.

## The Cornerstones of the Ischemia-Reperfusion-Injury Cascade

**Figure 1:**
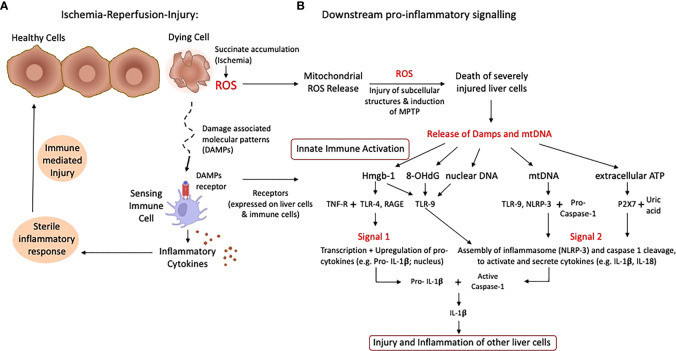
Schematic presentation of ischemia-reperfusion-injury with pro-inflammatory signaling. The rapid succinate metabolism at complex II leads to the initial key event of ROS release when oxygen is reintroduced into ischemic tissues. Based on the level of accumulated succinate during ischemia, a number of cells are severely affected and die with subsequent release of mitochondrial DNA and Damps **(A)**. Specific receptors are expressed on liver and immune cells, which trigger transcription and upregulation of pro-cytokines, creating an inflammatory milieu. Various Damps molecules instigate proinflammatory signals and the assembly of the inflammasome (NLRP-3) plus caspase cleavage, which activates available pro-cytokines **(B)**. Such features lead to the injury of previously less activated and affected cells, which releases additional damps and cytokines. ATP, Adenosine-trisphosphate; Damps, danger associated molecular patterns; Hmgb-1, High mobility group box protein-1; IL, Interleukin; ROS, reactive oxygen species; TLR, toll like receptor.

Ischemia-reperfusion injury (IRI) is a paradoxical cascade of injury, which occurs during re-oxygenation of an ischemic organ (11, 12). It is now well recognized, that the predominant effector of this injury is an early burst of reactive oxygen species (ROS), occurring across numerous types of tissues, including liver, kidney, lung, heart, muscle, and brain (7, 13–17). While there are several sources of cellular ROS, for example the xanthine oxidase pathway or NADPH oxidase systems, superoxide production during IRI is reported to be the result of a dysregulation of the electron transport chain, with electrons leaking at various sites when oxygen is re -introduced following a period of ischemia (18, 19). The level of previously accumulated succinate, the most important Krebs cycle metabolite in this context, plays a crucial role and associates with the level of released ROS from mitochondrial complex I (20). With prolonged cellular ischemia, succinate accumulates to a higher extent, which also correlates with the energy content of liver cells (11). When mitochondrial succinate levels are high, they trigger an immediate metabolism of this detrimental compound by mitochondrial complex II and the TCA-cycle as soon as oxygen is reintroduced and the respiratory chain reestablishes an electron flow. This is however initially uncoordinated and retrograde and therefore triggers the release of reactive oxygen species (ROS) at complex I (11, 18). Cells, which are severely affected by ROS may die and release further molecules, including Danger-associated molecular pattern (Damps) and cytokines, which trigger the inflammatory response from other surrounding cells, initially less affected (**Figure 1**) (8). Such Damps are released from all severely injured liver cells in combination with mitochondrial DNA and represent the first level of innate immune system activation (9, 11, 21–23). With the presentation of surface markers on activated macrophages, recipient T cells are attracted and activated, which represent the next level of contribution to the innate immune response (7, 8, 24). The next subchapter provides further details on specific innate immune signaling among involved cells.

## Mechanism of Innate Immune Signaling

**Figure 2:**
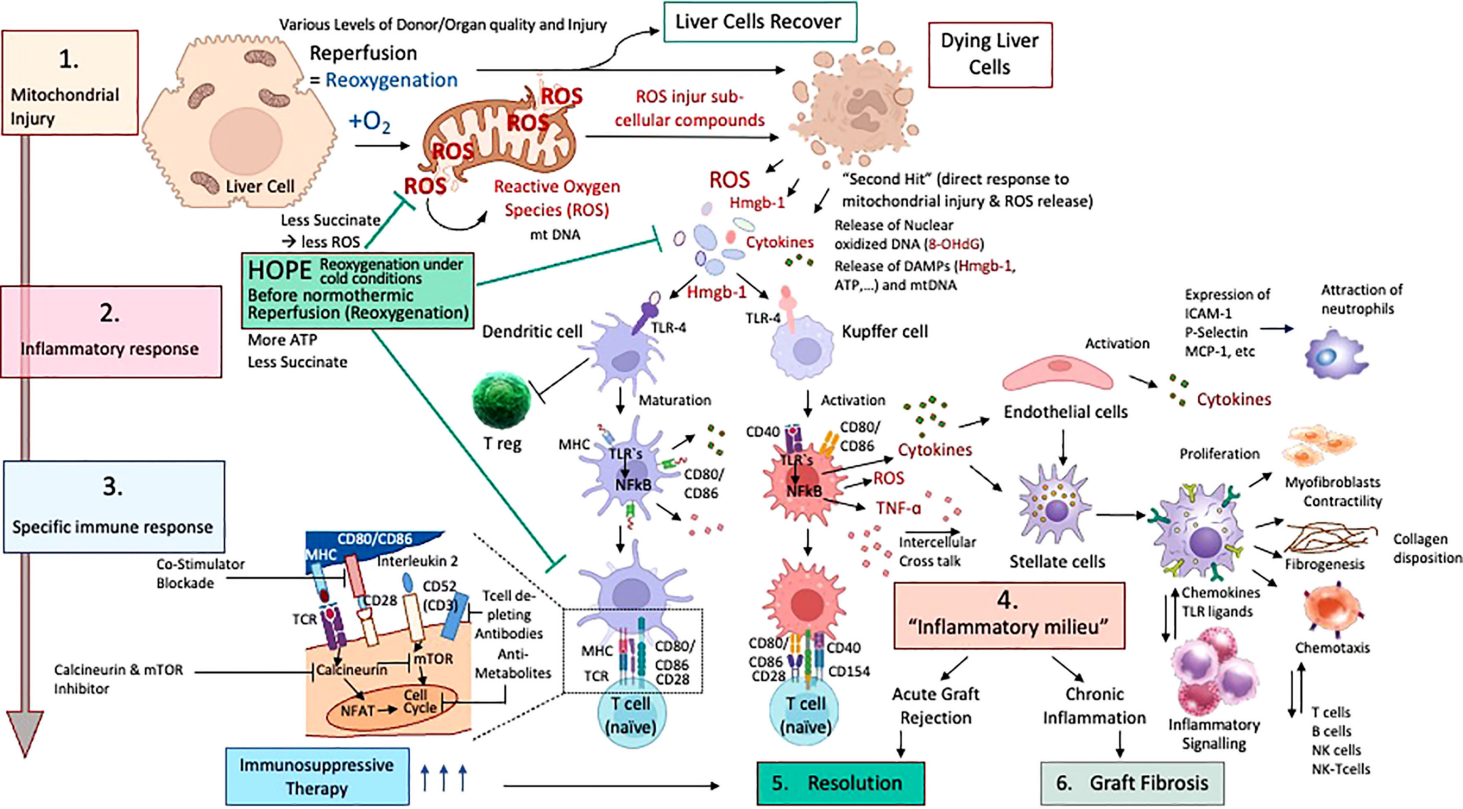
Cascade of ischemia-reperfusion-injury with specific downstream activation of the innate immune system; Different stages of ischemia reperfusion injury (IRI) with innate immune activation and resolution with or without pharmacological treatment in liver transplantation are presented. According to the accumulated succinate, ROS are released at reperfusion (1) with subsequent release of mitochondrial DNA and Damps molecules, which link IRI to innate immune response. (2) Subsequently, dendritic cells and macrophages become activated and present their well-known surface receptors to circulating T cells, which become in turn activated (3). Recipient neutrophils and other immune cells are also attracted by activated endothelial cells in the organ. With the involvement of other liver and recipient immune cells, including stellate cells and fibroblasts, T and B cells plus killer cells, the inflammatory milieu achieves a chronic stage (4) and is resolved by pharmacological upstream treatment (5). Chronic processes may also become very advanced and severe with subsequent fibrosis of the implanted organ (6) if resolution mechanisms fail.

Mitochondrial ROS are a consequence of mitochondrial disruption in all liver cells, macrophages and T cells. Once oxygen is reintroduced after a period of ischemia, mitochondrial electron transfer complexes I and III serve as the major sites of ROS production (18, 25). Released ROS are directly antibacterial, but also signal the release of inflammatory cytokines. Following their release, ROS molecules first injure subcellular structures within the same cell, which releases further proinflammatory molecules (26). Cells severely affected by the cascade of ischemia-reperfusion-injury (IRI) leak pro-inflammatory molecules, which in turn activate other cells, initially less affected (**Figure 1A**). Such pro-inflammatory molecules include the release of damage-associated molecular patterns (DAMPs), which activate local antigen presenting cells (21). DAMP receptors are toll-like-receptors (TLR) or receptors for advanced glycation end products (RAGE), which mediate downstream cytokines release with subsequent production of more ROS by non-parenchymal liver cells, mostly Kupffer cells. ROS and DAMP release promotes two main signals, first transcription and upregulation of pro-cytokines in the nucleus (e.g., IL-1β) and second, the assembling of the inflammasome (NLRP-3) and capsase-1 cleavage to finally activate and cleavage such pro-cytokines (**Figure 1B**) (8, 27, 28). TLR-signaling also involves multiple adaptor proteins MyD88, TIRAP, TRIF, TRAM, and the activation of the transcription factors NFκB, AP-1 and IRF3, which ultimately lead to a maturation of dendritic cells with consecutively presentation of antigens together with the expression of co-stimulatory molecules, which attract immune cells (**Figure 2**) (21, 29, 30).

T cells appear as main contributors in this context and become activated (T cell receptor signaling), thereby inducing a further increase in mitochondrial ROS production (31, 32). Interestingly, T cell subtypes that lack the Complex-III component RISP are more protected from ROS release. Subsequently, such T cells have a decreased expression of the activation markers CD-25 and CD-69 (31). Pharmacological blocking of this T cell activation process antagonizes their IL-2 production. One subtype of such immune cells appear as regulatory T cells, which are described with a reduced expression of CD-25 and subsequent less IL-2 production. Such T regs are therefore protective from IRI-injury and may help to reduce inflammation (33). The antagonism of CD-25 in Treg deficient mice was shown to protect murine kidneys from IRI-injury (34). Despite such known protective effect, the role of Tregs was described controversial in different stages of brain IRI. While Tregs were found to reduce inflammation and promote tissue repair in the later stage of resolution after IRI, during the acute phase stroke pathogenesis was rather pronounced by Treg (34). Additionally, in other models as for example hindlimb ischemia and reperfusion, Treg depletion through anti-CD25 led to higher levels of inflammation and to a higher number of newly developed vessels.

TCR signaling also leads to the release of calcium, stored in the endoplasmic reticulum, which can be taken up by mitochondria to drive the Krebs cycle enzyme activity, thereby increasing the amounts of NADH and Krebs cycle intermediates, including succinate (35). Mitochondrial calcium influx contributes to mitochondrial ROS generation in T cells, and succinate drives further mitochondrial ROS generation in macrophages (31).

Mitochondrial ROS also function as a signal in B cell activation. B cell activation by antigen and helper T cells induces several processes such as somatic hypermutation, to diversify the B cell receptor for antigen, and class-switch recombination (CSR), which enables B cells to express a particular type of immunoglobulin (36). The ligation of the BCR stimulates calcium release into the cytoplasm, which promotes ROS production. Stimulation with LPS and IL-4 or with anti-CD40 and IL-4 generates a population of B cells with increased mitochondrial mass, membrane potential and ROS production compared with naive B cells (37). Undifferentiated cells within this population undergo CSR after differentiation. Mitochondrial ROS may also act to induce CSR by inhibiting the synthesis of heme, a molecule that decreases CSR by antagonizing BACH2, a key transcription factor for CSR57 (36).

In summary, these observations indicate that T and B cells require ROS production for the generation of an appropriate immune response. Innate immune cells are similarly reliant on ROS production, with Krebs cycle intermediates such as succinate, citrate, fumarate and itaconate emerging as important regulators of the production of this noxious agent, as well as other events (38). Overall, mitochondrial ROS, originating from a variety of mitochondrial sources, are key signaling molecules for immune-cell function.

## Succinate as Regulator of IRI-Associated Inflammation

During ischemia, various Krebs cycle metabolites with important inflammatory signaling function accumulate in all involved cells. These metabolites are present because of the occurrence of break points in the Krebs cycle during ischemia. The interruption at the enzyme succinate dehydrogenase (SDH), which represents mitochondrial complex II, and converts succinate to fumarate, is associated with an accumulation of succinate, a metabolite with proinflammatory activity and roles in immunity (18).

Mitochondrial succinate, once accumulated to a significant extend, triggers two main metabolic features at reoxygenation. First, when high succinate levels are present, mitochondria aim for a rapid metabolism and immediate reestablishment of the electron flow, which is however reverse (RET) with subsequent ROS production from complex I, thereby instigating the inflammatory IRI-cascade (**Figure 1**, **2**) (11, 39, 40). Succinate oxidation by SDH, also leads to HIF-1α activation and HIF-1α-dependent gene expression; a notable example of a gene affected in this way is IL1B, which encodes IL-1β (41). Succinate also limits the production of anti-inflammatory cytokines, particularly of IL-10. Inhibition of SDH with dimethylmalonate (DMM) profoundly inhibits LPS-induced ROS generation and the expression of IL-1β and a range of proinflammatory genes in macrophages, and boosts the expression of IL-10 and anti-inflammatory genes (8, 10, 24, 42). DMM administration in mice treated with LPS results in decreased IL-1β expression and a boost in IL-10 expression systemically (31, 35). LPS treatment boosts the mitochondrial membrane potential in macrophages, which is dependent on increased glycolytic ATP production. Limiting of LPS-induced membrane hyperpolarization, which is required for RET, inhibits IL-1β induction in response to LPS in macrophages (31). The induction of IL-1β by LPS and succinate is also impaired in macrophages, that express the enzyme alternative oxidase (AOX), which limits RET. All of these observations not only confirm a role for mitochondria-derived succinate as a critical proinflammatory mediator, but also shed light on the mechanism by which LPS drives ROS production, and provide further rationales for why macrophages favor aerobic glycolysis and decrease Oxidative Phosphorylation (OXPHOS)

in response to LPS. These metabolic alterations repurpose mitochondria from ATP synthesis to ROS production to promote and maintain a proinflammatory state (18, 21, 23, 25, 43). An antibacterial role of SDH was also suggested by the observation that the inhibition of SDH during bacterial infections rendered mice more susceptible to infection. Taken together, these findings identify complex II (SDH) as one key control point IRI-associated inflammation.

## Mitochondrial Signaling Activates NLRP-3 Inflammasome

Mitochondrial antiviral signaling protein (MAVS) is another key signaling protein activated by the different RNA sensors, including RIG-I and MDA5 (29). It in turn activates pathways, that regulate the transcription factor NF-κB and IRFs to promote proinflammatory gene expression (8, 29, 44, 45). Interaction with the outer mitochondrial membrane is essential for a fully functional MAVS molecule. Mitochondrial ROS can induce and drive MAVS oligomerization, leading to the production of type I interferon, independent of RNA sensing, which in turn suggests that MAVS might be a key sensor of mitochondrial ROS, that acts to promote the recipient defense and inflammation (25, 29, 31). Furthermore, MAVS associates with Inflammasome (NLRP-3) and promotes its oligomerization, which leads to caspase-1 activation (8, 23, 43, 46–48). Activation of NLRP-3 with the synthetic Toll-like-receptor (TLR) -7 ligand imiquimod has recently been shown to occur as a result of the mitochondria ROS production from complex I and the quinone oxidoreductase NQO_2_ (31, 48). This effect was independent of potassium efflux, which highlights the importance of mitochondrial ROS for NLRP-3 activation (49, 50). Finally, NLRP-3 is also regulated by cardiolipin, a lipid of the inner mitochondrial membrane, which translocates to the outer membrane, where it recruits NLRP-3 after mitochondrial membrane depolarization (31). This interaction appears crucial for NLRP-3 activation, and suggests that mitochondria function as signaling hubs and activate the innate immunity. NLRP-3 activation in turn leads to mitochondrial damage and subsequent mitochondrial ROS release, which represents a feedback loop between NLRP-3 and mitochondria (23, 48). Mitochondria are therefore critical for signaling by the described three major innate immune signaling pathways.

## Mitochondria: The New Therapeutic Target?

**Figure 3:**
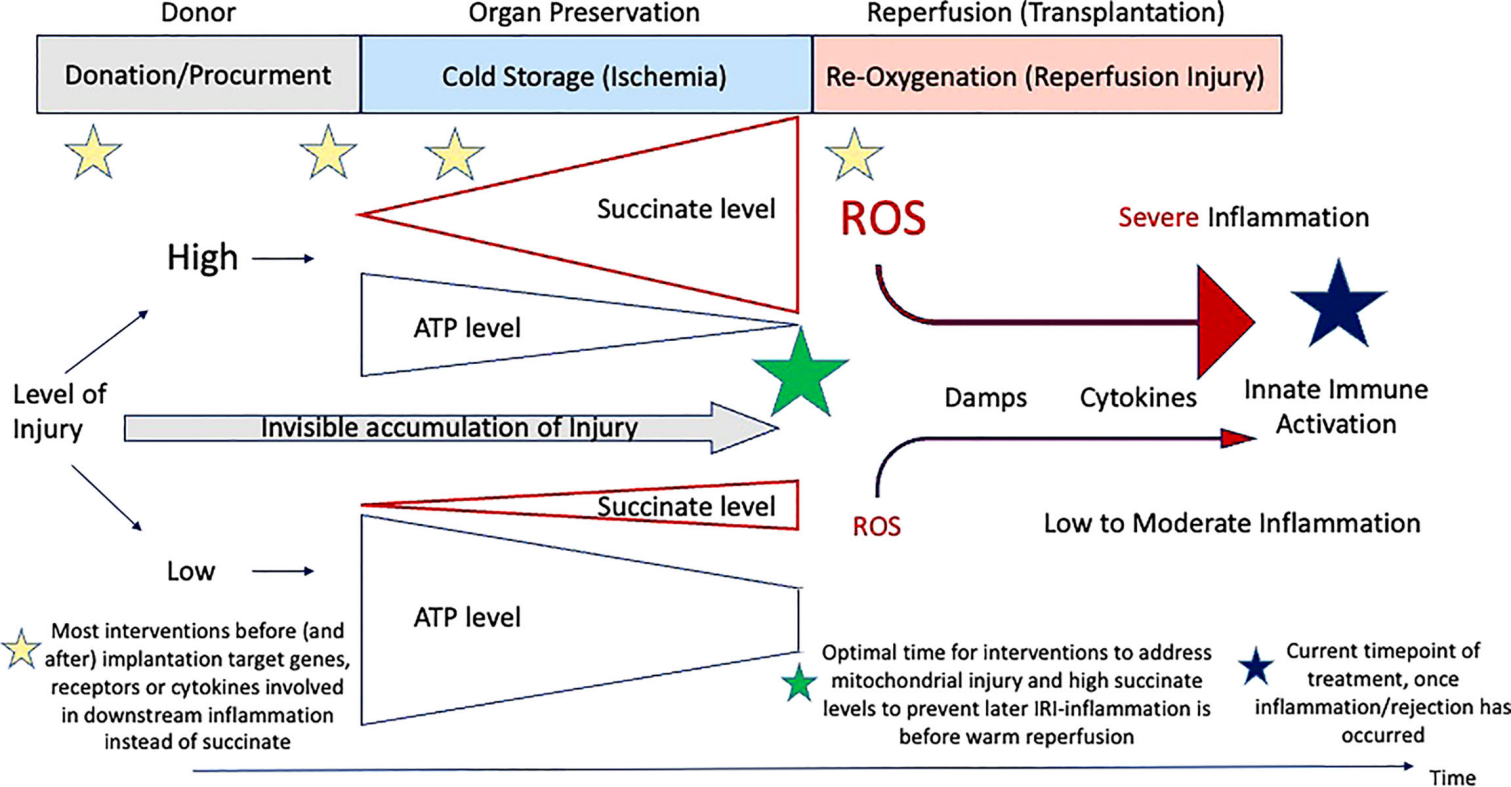
Timing of treatment modalities to address IRI-associated inflammation. Overview on the current timings when therapeutics are administered from the donor to implantation. Most molecules target genes or downstream receptors beyond the instigating processes, instead of succinate and subsequent ROS release.

A relatively novel strategy to decrease the posttransplant inflammation and the need of IS is to approach the crosslink between organ ischemia-reperfusion injury (IRI) and immune response. Instead of focusing on the key instigator of the IRI-cascade in mitochondria, most teams target the suppression of proinflammatory molecules, released in response to ROS later after reperfusion (**Table 1**). Others focus on the suppression of defense mechanisms. Preventive or therapeutic measures with impact on IRI-associated inflammation were applied for example in the donor as direct treatment or as an additive to the donor flush solution (56). Others have added specific molecules during cold storage or as a treatment in the recipient. Most molecules were however not yet transferred into clinical practice, although the results seen in experimental studies appeared quite promising with impact on IRI-associated inflammation, the effect got either lost or was limited when explored in transplant models (40, 57, 58). The reason behind could be the target of too peripheral individual genes or molecules of the IRI-cascade. Although the underlying mechanisms of IRI are increasingly linked to tissue succinate accumulation during ischemia with subsequent ROS release after reoxygenation, this key metabolite is rarely addressed in studies (40). A significant protection from IRI-associated complications could be achieved through the prevention of succinate accumulation before or during donation (e.g., before ischemia) or also by a slow succinate oxidation before normothermic reperfusion at transplantation (40). Already established succinate levels could be eliminated through a slow activation of complex II, before reintroducing oxygen at normothermic temperatures (**Figure 3**) **(** 40). Reduced cellular succinate stores prevent the initial burst of ROS release, which therefore eliminates the initial danger event of the IRI- cascade (40). In addition to succinate, other compounds in mitochondria could be the target to reduce IRI. Malonate could for example be used to block complex II (SDH) and limit succinate accumulation and also impact on the rapid succinate oxidation at reperfusion (11, 18, 38). Next, the inhibition of SDH with DMM may also provide a therapeutic benefit by limiting ROS production and proinflammatory responses, and boosting the anti-inflammatory response (19, 31). If the reprogramming would sustain, it could also offer the possibility of inducing remission in chronic inflammatory diseases. In addition, the blockage of the mitochondrial permeability transition pore (MPTP) with Cyclosporin, as tested in mice, could be advanced into human practice. However, despite promising results of a randomized controlled trial (RCT), the effect of this pharmaceutical was limited in a recent phase III study in hearts (59, 60).

**Table 1:** Clinical studies with the impact of machine perfusion on immune response within the last 3 years.

Authors, Year study type & Country	Number and Type of livers	Type & Duration of Donor warm ischemia time (min)	Duration of cold ischemia before perfusion	Duration of Perfusion	Duration of Follow-up	Main Findings	Discussion
Clinical studies with the impact of hypothermic machine perfusion on the immune system							
Van Rijn et al, 2021, Randomized controlled trial, Europe (51)	78 DCD livers each arm (D-HOPE vs. CS)	Total DWIT cDCD D-HOPE: 29 (IQR: 22-33); CS group: 27 (IQR: 21-35) Asystolic DWIT cDCD D-HOPE: 11 (IQR: 8-13); CS group: 11 (IQR: 8-15),	6hrs 11min (IQR: 5hrs 16min – 6hrs 55min)	2hrs 12 min (IQR: 2hrs – 2hrs 33min)	6 months	D-HOPE reduces acute rejections: D-HOPE 11.5% vs cold storage control 20.5%	Follow up of 6 months
Czigany et al, 2021, Germany (+Prague) (52)	23 DBD livers each arm (HOPE vs. CS)	None	DBD HOPE: median 6.3hrs (IQR: 5.2-7.8hrs); DBD SCS: median: 8.4hrs (IQR: 7.8-9.7hrs)	DBD HOPE: median 2.4hrs (IQR: 1.7-3.4hrs)	12 months	HOPE treatment reduced the acute rejection rate from 26% (CS control) to 17% (HOPE group), primary endpoint is reduced Peak ALT levels (p=0.03), other endpoints: shorter ICU (p=0.045) and hospital stay (p=0.002), less major complications ≥ Clavien Grade IIII (p=0.036), cumulative complications (CCI: p=0.021), estimated costs (p=0.016)	Study was not powered for complications, DBD grafts
Retrospective Studies							
Ravaioli et al, 2020, Italy (53)	Extended DBD/HOPE=10, SCS controls (n=30) None	None	14.5hrs (IQR: 10.8-22hrs)	2.2hrs (IQR: 1-3.5hrs)	12 months	Tendency toward a lower ACR rate: 10% HOPE group and 13.3% CS control; No PNF and lower rate of EAD, lower recipient transaminases after HOPE treatment and 100% graft survival compared control,	Low case number, matched cohort study, DBD
Schlegel et al., 2019, UK, Switzerland (54)	cDCD/SRR/HOPE=50; DBD/SCS=50 (control), cDCD/SRR (unperfused)=50	Total DWIT HOPE: median 36 (IQR:31-40); SRR: 25.5 (IQR:21-31); Functional DWIT HOPE: median 31 (IQR:27-36); SRR; median 17 (IQR:15-19); Asystolic DWIT HOPE: median 19 (IQR:17-21); SRR: median 12.5 (10-15)	cDCD/HOPE: median 4.4hrs (IQR: 3.5-5.2hrs); SRR group: 4.7hrs (IQR: 4.3-5.3hrs)	Median 2hrs (IQR: 1.6-2.4hrs)	5 years	cDCD/HOPE with less acute rejection; 4% HOPE group, 28% CS group, p=0.0019, SRR DCD: 22% (n=11/50) with 10% (n=1/69) graft loss; HOPE: 8% (n=4/50) with 0% graft loss; Less PNF, HAT and ischemic cholangiopathy result in an improved five-year survival of HOPE treated extended DCD liver grafts	Matched cohort study, retrospective
Patrono et al, 2019, Italy (55)	Extended DBD/D-HOPE, macro-steatotic=25, DBD/SCS=50 (control)	None	311min ±53 (mean, SD)	186min ±49 (mean, SD)	6 months	Lower rate of acute rejections with 8.6% (HOPE group) and 16% CS control, lower rate of post-reperfusion syndrome, acute kidney injury grade 2-3, and EAD, lower rates of anastomotic strictures: CS: 12% (n=6/50); D-HOPE: 16% (n=4/25), SCS: 8% (n=4/50), 2 symptomatic patients; D-HOPE: 8% (n=2/25), both asymptomatic	DBD grafts

Studies are summarized according to the literature within the last 3 years concerning transplantation of controlled DCD or DBD livers procured with standard cold storage and machine perfusion, included were studies with a cold storage control group, either DCD or DBD and with information on acute liver rejection or other parameters relevant for the immune response; ACR, acute cellular rejection; DBD, donation after brain death; DCD, donation after circulatory death; DWIT, donor warm ischemia time; EAD, early allograft dysfunction; HOPE, hypothermic oxygenated perfusion; IQR, interquartile range; SCS, standard cold storage; SRR, super rapid retrieval; concerns DCD donors.

Various other molecules and drugs are currently developed to be used to reduce downstream inflammatory processes induced by ROS, Damps and mitochondrial DNA. Most studies appear however older and involve only animal models with only very few that have explored the effect in a transplant model (56).

## RNA Interference as Tool to Reduce IRI-Associated Inflammation

Next, the natural process of RNA interference (RNAi) with silencing of specific genes was recently explored and introduced into transplantation settings. Various experimental studies were presented with the interference of the specific RNA, targeting genes of IRI-injury and immune activation. Unfortunately, in most studies, RNAi is explored in the donor 1 to 72hrs before ischemia (61). This approach appears however rather unpractical in clinical settings, where donor treatment is prohibited in most countries (62). Only very few preclinical studies involve other treatment routes, including administration through portal vein infusion (61, 63).

In addition to RNAi other molecules, including antioxidants and antibodies are administered to block specific molecular reactions. Li et al. added the CD47m antibody into the liver flush solution during donation. CD47 is a member of the immunoglobulin superfamily and is involved in various pathways related to ROS molecules. Recipients were found with lower rates of acute rejection and a lower severity in this allogenic mouse model (64).

In summary, various treatments are currently explored, frequently targeting rather peripheral processes instead of mitochondrial components. Multiple routes are used, including donor injections, organ flush and recipient treatment. The modulation of the current standard cold storage preservation, with the implementation of machine perfusion technologies, might serve as most attractive tools to supply pre-injured organs with beneficial compounds before implantation in the future. However, prior to successful utilization of machine perfusion as delivery tool, underlying mechanisms of protection and injury should be explored and understood.

## The Role of Organ Perfusion Techniques for Immune Modulation

Novel organ perfusion strategies appear of interest and various groups have promoted the technological development in the last decade (51, 54, 55, 65–68). Despite this advancement, available data on the solitary impact of machine perfusion (MP) on ROS-mediated allograft oxidative injury and subsequent immune response remain scarce (5, 42).

Novel organ perfusion approaches can be divided into two main strategies, first in-situ perfusions, which include regional perfusion, mainly done at normothermic temperatures and secondly, ex-situ techniques, where organs undergo perfusion on a tailored device either after cold storage (e.g., endischemic) or instead (69). Normothermic regional perfusion (NRP) is applied in the donor immediately after circulatory death (DCD). Leading countries, where this technology is routinely used in DCD liver donors include Spain, France, Italy and a few centers in the United Kingdom (70–73). The early graft evaluation during NRP in the donor helps to select livers with too high injury. This selection is mainly based on the macroscopic liver appearance, the perfusion quality, pH, liver transaminases and lactate measured from the NRP-circuit. DCD liver preserved with NRP were shown to achieve immediate function and recipients experienced reduced levels of biliary complications after transplantation (68, 74). With the overall aim to push regulatory and donor risk boundaries, the limitations of all perfusion techniques are increasingly described. With NRP, DCD livers were well preserved provided that the national regulations of donor and recipient risk were respected. French centres for example described a higher number of graft loss with the use of NRP-treated grafts when the donor warm ischemia time or recipient lab MELD exceeded national criteria (72). With additional cold storage time following NRP and liver procurement, the exact metabolic situation of such organs appears not well known at the time of implantation (69). In this context, prolonged cold ischemia of more than 7hrs was recently described as risk factor for graft loss in Spain, when combined with NRP (75). This risk was even more pronounced when such DCD grafts were implanted into recipients waiting for a retransplantation (75). Comparable to any other technique of normothermic reperfusion, where oxygen is reintroduced into ischemic tissue, during initial NRP, the cells and more specifically mitochondria have the main goal to metabolize high succinate concentrations rapidly with subsequent ROS release from mitochondrial complex-I and downstream inflammation. This has been recently demonstrated in the setting of clinical NRP in Italy with the quantification of cytokine levels in the recirculating NRP-perfusate (76). Based on the known link between IRI and innate immune activation, more data are required to describe the impact of NRP on the innate immune system, and ideally in context of an increased utilization of risky donors.

In contrast, two main ex-situ machine perfusion techniques are currently explored (51, 67, 68). First, the normothermic machine perfusion (NMP) technique using a blood-based perfusate at 37°C, which is ideally applied after liver procurement and instead of cold storage, replacing cold ischemia with a subsequent reduction of liver injury and IRI(62). And secondly, a logistically less challenging perfusion strategy, applied after cold ischemia in the recipient centre using a hypothermic oxygenated perfusion (HOPE) prior to implantation (11, 77). Of note, although various groups have explored the use of such perfusion techniques as delivery tool for molecules or stem cells in ex-vivo models of liver perfusion, the underlying mechanisms of such perfusion techniques are not well enough understood and deserve more recognition (78).

Perfusion techniques done at warm temperatures were found to trigger the same ROS-induced inflammation as seen with the IRI-cascade after liver transplantation. The level of IRI might however be dependent on the perfusate components. The literature describing such features of liver NMP in a transparent way is very scarce (28, 79, 80). Normothermic perfusion strategies induce an inflammatory environment with the release of ROS and DAMPs and consecutive activation of toll-like-receptors (28, 81). Downstream activation of the innate immune system after NMP of livers can be likewise expected, promoting acute organ rejection, particularly when NMP is applied after a relevant period of standard cold storage (82, 83). Recent data from normothermic kidney perfusion support such mechanistical insights (84).

Unfortunately, markers of immune response are frequently not reported in clinical and experimental studies with normothermic perfusion. We were therefore also not able to add clinical studies to **Table 1**, which assess the impact of NMP on the immune system.

Jassem et al. have recently compared the expression of immune-related pro-inflammatory genes between cold stored livers (n=12) and grafts that underwent NMP instead (n=27) from brain death donors (DBD) (85, 86). Cold stored livers showed a higher expression of genes involved in innate immune activation, neutrophil chemotaxis and platelet activity with higher levels of recipient plasma cytokines (85). Livers, exposed to NMP demonstrated less amounts of specific T cells, known for their production of Interleukins 2, 4 and 17, and Interferon y. The proportion of regulatory T cells was found slightly higher in the NMP group, particularly the tissue-resident subtype CD4^pos^CD25^high^CD127^neg^FOXP3^pos^ was described with higher concentrations throughout prolonged normothermic perfusion (85). Of note, the replacement of cold storage by NMP led to a lower liver injury in the NMP group, while grafts in the control group were exposed to the entire duration of cold ischemia before implantation and exploring the immune response presented here (67, 85, 87). The work by Scheuermann et al. presents one of the very few studies actually quantifying the inflammatory injury during NMP, compared to other perfusion techniques. Authors have demonstrated that liver perfusion at lower temperatures, e.g., subnormothermic, induces less inflammation compared to NMP (88).

**Figure 4:**
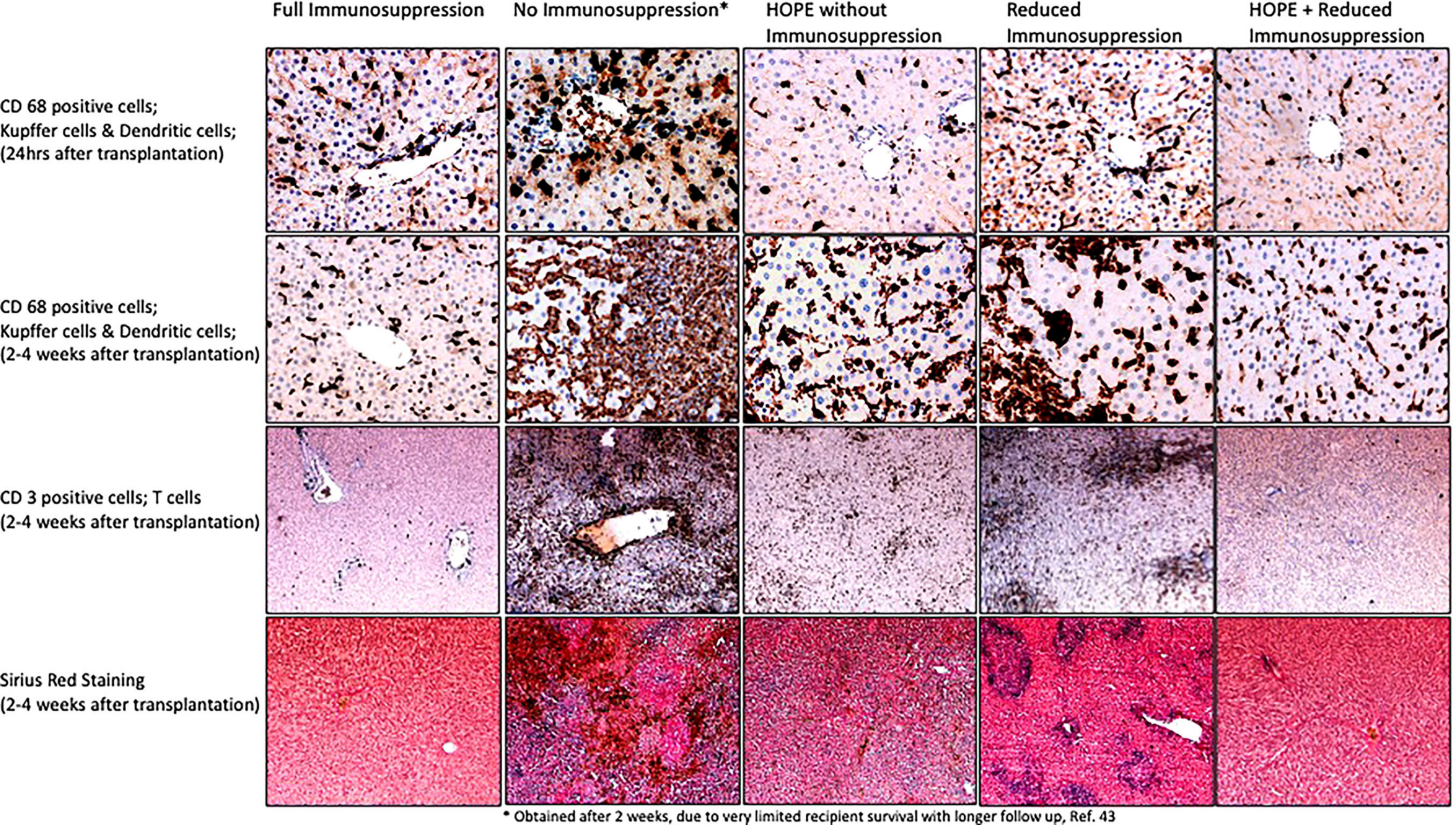
Impact of HOPE on innate immune response after allogeneic liver transplantation; To address the accumulated succinate with a slow oxidation at complex II is a key mechanism to avoid the massive ROS release and subsequent IRI cascade with complications after transplantation. Machine perfusion is therefore a well explored new method to improve and assess metabolic processes. Hypothermic oxygenated perfusion (HOPE) before implantation was shown to reduce the accumulated succinate and to improve complex I and II function. Using a model of allogeneic liver transplantation, the protective effect of HOPE on the innate immune system was demonstrated with a lower number of activated of Kupffer cells (through less Damps release) and subsequently a lower number of infiltrating T cells in transplanted livers, compared to untreated controls (without HOPE and without immunosuppression). Of note, HOPE treatment achieved the best protection from Kupffer cell and dendritic cell activation early after implantation, e.g., at 24hrs, also compared to the group with full dosage of immunosuppression, which requires time until the most effective blood levels are seen. The protective effect of HOPE was still present 4 weeks after implantation, although the delayed immune response became visible. HOPE treatment was therefore combined with a low dose of immunosuppression, which led to acute rejection when applied alone. HOPE with reduced immunosuppression protected recipients from innate immune activation and acute rejection, similarly to recipients which received the full dose of tacrolimus (immunosuppression). These images were obtained from samples from the study presented in reference (42) (samples and histological images were not published before in this reference). CD, cluster of differentiation.

In contrast, hypothermic oxygenated perfusion (HOPE), is performed at 6-10°C using an artificial perfusate, which is highly oxygenated (60-80kPa) (89, 90). HOPE therefore was shown to reprogram mitochondrial during the introduction of oxygen at such low temperatures. During HOPE the electron flow is reestablished with subsequent steady and slow metabolism of accumulated succinate and ATP reloading at the same time. While the respiratory chain is functioning the high load of NADH at complex I is also reduced by metabolism to NAD, which is a surrogate marker of a functioning complex I and energy recharge (91). Mitochondria, which underwent reoxygenation at hypothermic temperatures are switched and can handle the reperfusion with blood at normothermic temperatures without thew known detrimental rapid succinate metabolism and with much lower ROS production (11, 81, 92, 93). HOPE-treatment achieves this by increasing the activity of mitochondrial complex proteins (11). Based on these significant changes during HOPE, mitochondria are prepared and experience less oxidative stress and subsequently trigger less Damps release with less TLR activation and a reduced innate immune response (28, 79). Ex-vivo graft treatment by HOPE was consecutively also found to prevent downstream T-cell activation in allogenic liver and kidney transplant models, without any additional immune suppressive treatment (17, 42). Combined experimental protocols allowed to reduce the immunosuppression to one third of the normal dosage when IS was combined with HOPE. An excerpt of the results found after transplantation with various IS and HOPE combinations in this allogeneic liver model is shown in **Figure 4** (42). Normal livers from Lewis rats were procured and with minimal cold ischemia transplanted into Brown Norway recipient rats, which is well-described in the literature as a known model of allogeneic liver transplantation. Of note, recipients without any immunosuppression achieved poor survival, expectedly shorter than two weeks in most. In contrast, HOPE treatment in these allogeneic rodent livers led to similar survival rates as seen in recipient, which received the normal immunosuppressive treatment with Tacrolimus (42). In combination with a reduced Tacrolimus dosage, allogeneic liver recipients achieved prolonged survival when combined with HOPE treatment. Of note, similar findings are described in the literature for allogeneic models of kidney transplantation using endischemic HOPE (17). These findings from preclinical studies are now also paralleled by an increasing body of clinical studies with and without a randomized design. **Table 1** summarizes clinical studies reporting an impact of HOPE on the immune activation after human liver transplantation. The recently presented RCT from the Groningen group found as a secondary endpoint a 9% reduction of acute rejections after DCD liver transplantation with dual HOPE treatment compared to cold storage controls (51). A similar tendency was described by another RCT from Germany, where the team described a reduction of acute rejections from 26% in the cold storage study arm to 17% after HOPE (52). Earlier retrospective studies have already demonstrated similar results. The Zurich group compared the effect of HOPE in extended DCD liver grafts with unperfused, cold stored controls from the United Kingdom. Of note, livers with HOPE presented an acute rejection in 4%, which is a significant reduction from 28% seen in unperfused controls (p=0.0019)(**Table 1**) **(** 90). Although the impact of machine perfusion on costs is not well enough explored yet, a more than 10-20% reduction of acute rejections will most likely reduce transplant related costs with less requirements of liver graft biopsies and readmissions. In addition to the impact on biliary and overall complications, further results are awaited with regard to costs. Another important factor is the perfusion duration of HOPE. A recent multi-centre study has collected outcomes of 93 human liver transplants (50 DCD, 43 DBD) with a prolonged HOPE treatment of >4hrs and a median overall preservation of 10hrs. Outcomes were excellent and comparable to other series reported with shorter endischemic HOPE (94). Of note, prolonged HOPE of >4hrs was also shown after more than 10hrs of cold storage in extended criteria donor livers in Germany and in combination with high MELD recipients in Brasil (95). A prospective study on prolonged HOPE is currently ongoing in the Netherlands to confirm the safe prolongation to compensate logistical issues (96).

## Machine Perfusion as a Tool to Deliver Specific Molecules

**Figure 5:**
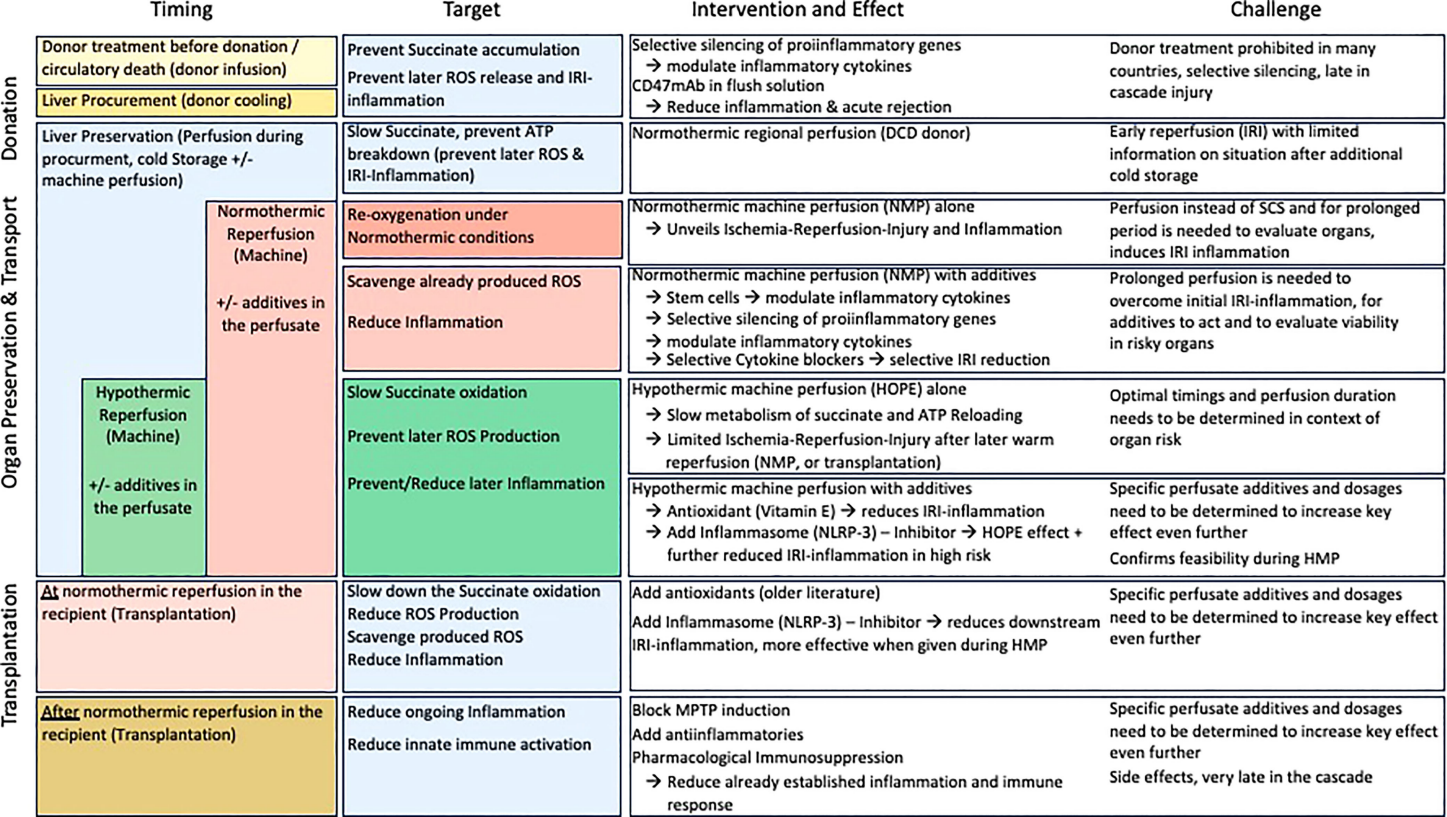
Therapeutic strategies to target ischemia-reperfusion injury and improve outcomes after liver transplantation; This figure provides an overview of currently applied pharmacological and non-pharmacological modalities with impact on IRI associated features. From the donor, procurement to preservation and reperfusion (transplantation) modalities, their targets and results as well as challenges are presented. The majority of strategies affects individual genes or receptors, which might lead to an even higher proinflammatory response by other genes not affected.

Recirculating perfusates may provide two main benefits during machine perfusion. First, injured and dying cells release specific molecules, which might signal functional deficits and could be used to assess organ viability (97). And secondly, perfusate could be used to carry specific molecules into the cells and subcellular compounds to prevent production and release of IRI-associated compounds. Various therapies, such as pharmacological agents, genes, stem cells and nanoparticles were administered into organs through this route in preclinical studies (56, 57). The technique of normothermic perfusion is more frequently applied because of the assumed better transport and uptake of such molecules at 37°C, when compared to hypothermic perfusion settings. This last subchapter describes studies within the last 3 years, where machine perfusion was used to deliver compounds with an impact on the innate immune response (**Table 2**). Only very few studies explore the impact in a transplant model. Cao et al. exposed DCD livers with 30 minutes of donor warm ischemia time to 4hrs of NMP with the addition of bone marrow-derived mesenchymal stem cells, which were found to inhibit the release of Hmgb-1 with subsequent reduction of TLR-4 activation. Authors demonstrated a clear effect on the early innate immune response through NMP with such stem cells compared to unperfused cold stored controls (98). This work was paralleled by Yu et al, who blocked NLRP-3 in a pig DCD liver transplant model. All experimental groups underwent hypothermic perfusion and the NLRP-3 blocker was administered either during perfusion or after implantation in the recipient. Groups with additional NLRP-3 blockage were found with lower innate immune response independent from the administration route compared to hypothermic perfusion alone. Of note the group where the recipient received the NRLP-3 blocker was superior to all other groups. This work demonstrated first, that the addition of molecules with a blocking effect on specific receptors is also effective during HMP or HOPE and secondly, that the already protective HOPE-effect on mitochondria can be even more enhanced with additional reduction of downstream inflammatory pathways (99). Other groups have for example demonstrated, that a selective cytokine blockage, e.g., IL-10 and TGF-β also reduces immune responses, however mainly in models with liver machine perfusion but without subsequent transplantation (100). Another approach is the addition of stem cells to perfusates. Laing et al. have added mesenchymal stem cells to NMP perfusates and show feasibility and comparable cell settling in perfused livers, when administered through different inflow vessels (78). Other agents, including defatting cocktails, were found to reduce the overall inflammation induced by NMP. Boteon et al. demonstrated an effect on the early innate immune response during NMP. Both, CD14, as found in macrophages and neutrophils, and pro-inflammatory cytokines were reduced by defatting cocktails in NMP perfusates (101). Another attractive approach is increasingly discussed with RNA interference. The group of Paulo Martins has recently demonstrated the first successful administration of siRNA during both, hypothermic and normothermic perfusion. The hepatocyte transfection was achieved through siRNA coating with lipid nanoparticles (102, 103). Of note, this is another evidence that such treatments are feasible in both perfusion approaches, warm and cold (53, 55, 103). The impact of such selective gene down regulation might however trigger responsive upregulation of other genes and subsequent cytokine and mediator release, where more studies also to identify the best pathways in humans are needed (**Figure 5**).

**Table 2:** Experimental studies exploring the impact of machine perfusion with or without specific perfusate additives on the immune system in liver transplantation.

Authors, Year study type & Country	Number and Type of livers, species	Donor warm ischemia time (min)	Duration of cold ischemia before perfusion	Type and Duration of Perfusion	Additives to Perfusate	Model of Liver Transplantation (yes/no)	Duration of Follow-up	Main Findings	Discussion
Experimental studies with liver perfusion and transplantation									
Schlegel et al, 2014 (42)	Rat livers, allogeneic model with full Tacrolimus, compared to HOPE without any Tacrolimus, and 1/3 of Tacrolimus with/without HOPE	n.a.	60min	1hr HOPE	none	Yes	4 weeks	HOPE protects from acute T cells mediated rejection, reduces T cell infiltration and CD40/CD86 expression, HOPE plus reduced IS was equally protective compared to full IS, lack of perfusate oxygen leads to the same injury as unperfused, untreated controls	B cell response was not addressed
Experimental studies with liver perfusion with the use of specific perfusate additives and subsequent transplantation									
Cao et al, 2020 (98)	30 rat livers, 5 groups of 6	30min	4 hours (SCS only)	4 hours NMP	BMMSCs and Heme-oxygenase 1 (HO-1)-modified BMMSCs	Yes	1, 7, 14 days	HO-1/BMMSCs combined with NMP exerted protective effects on DCD donor liver and significantly improved recipient prognosis. The effect of HO-1/BMMSCs was greater than that of BMMSCs and was mediated *via* Hmgb-1 expression and TLR-4 pathway inhibition.	Demonstrated the role of monocytes, requires further investigation needed on protective mechanism of BMMSCs, perfusion model can’t be translated into clinical practice
Yu et al, 2019 (99)	Pig livers, n=36, all DCD	30min	HMP + additive: 275 min HMP + post op additive: 268 min HMP + no additive: 274 min	HMP 2 hours all groups	MC950 (NLRP-3 Inhibitor)	Yes	3 days	The HMP-Postop group suffer the lightest ischemia reperfusion injury (IRI), and functioned best after transplantation. Model for the Early Allograft Function Score degree of injury in the hepatocytes and rate of apoptosis was lowest in the HMP-Postop group. The HMP-Postop group had the lowest downstream inflammation, and the level of IL-1β was lowest. Postop group functioned better than control group, but not comparable with HMP-Postop group.	Short follow up, unknown reference for dosage of additive
Experimental studies with liver perfusion and the use of specific perfusate additives without transplantation									
Carlson et al, 2021 (100)	22 Rat livers, 4hrs NMP vs. 4hrs SCS naiüve (n=4), CS (n=4), NEVLP (n=7), and NEVLP with anti-inflammatory cytokines (NEVLP-Cyt, n=7)	n.a.	SCS group only: 240 min	4hrs NMP at 37° (NMP and NMP+additive groups only)	IL-10 & TGF-β (20ng/mL)	No	n.a.	Pro-inflammatory gene expression during NMP, dominant in macrophages and dendritic cells, increased MHC II, CD40, CD86 expression, IL-10&TGF-β in NMP perfusates reduced immune activation	No transplant model, confirms induction of inflammation and immune system during NMP
Laing RW et al, 2020 (78)	6 Human livers, 2 DBD and 4 DCD	Not available	500 min	6 hrs NMP at 37°	MSC	No	n.a.	demonstrated that cells can be delivered directly to the target organ, prior to host immune cell population exposure and without compromising the perfusion. Transendothelial migration occurs following arterial infusion. MAPC cells appear to secrete a host of soluble factors that would have anti-inflammatory and immunomodulatory benefits in a human model of liver transplantation.	No transplant model, small sample,
Boteon et al, 2019 (101)	Hepatocytes and discarded human livers, n=10 2x (3 DBD + 2 DCD)	Treated group: 12min Control: 13 min	Median 737 min	12 hours NMP	Defatting cocktail	No	n.a.	Treatment reduced tissue triglycerides by 38% and macrovesicular steatosis by 40% over 6 hours Treatment down-regulated the expression of markers for oxidative injury as well as activation of immune cells (CD14; CD11b) and reduced the release of inflammatory cytokines in the per- fusate (tumor necrosis factor α; interleukin 1β)	No transplant model, Heterogeneous sample, higher risk in control group

N.A. means not applicable.

## Summary and Future Perspectives

Machine perfusion is an attractive tool to directly treat human livers prior to implantation and also to deliver specific molecules with impact on outcomes. The underlying mechanisms will however require more studies in the future to understand the individual impact of different perfusion strategies on the immune system. This review has mainly focused on the innate immune response, where an effect of HOPE perfusion is described in the current literature. The potential effect on the B cell response remains however entirely unknown. Most administered compounds during MP remain currently experimental and studies to identify the best route and dosage for administration are still lacking, particularly in humans. This is also valid for the spectrum of nanoparticles with various available types and formulations to be explored in the future.

## Author Contributions

RP, MC and AS designed the review and the figures. RP, MC, DD and AS: wrote the first draft. All coauthors discussed the content, revised the manuscript and approved the final version.

## Conflict of Interest

The authors declare that the research was conducted in the absence of any commercial or financial relationships that could be construed as a potential conflict of interest.

## Publisher’s Note

All claims expressed in this article are solely those of the authors and do not necessarily represent those of their affiliated organizations, or those of the publisher, the editors and the reviewers. Any product that may be evaluated in this article, or claim that may be made by its manufacturer, is not guaranteed or endorsed by the publisher.

## Abbreviations

ALT, Alanine Aminotransferase; AP-1, Activator Proteine-1; AS, Anastomotic strictures; AST, Aspartate-Aminotransferase; ATP, Adenosine-trisphosphate; BACH2, transcription regulator protein; CD, Cluster of differentiation; CIT, Cold ischemia time; CS, cold storage; CSR, Class switch recombination; Damp`s, Danger associated molecular pattern`s; DBD, donation after brain death; DCD, Donation after circulatory death; DMM, dimethylmalonate; DWIT, donor warm ischemia time; EAD, Early allograft dysfunction; ECD, Extended Criteria Donor; FMN, Flavin-mononucleotide; HA, Hepatic artery; GSH, Glutathione; HAR, hexa-ammineruthenium; HAT, Hepatic artery thrombosis; HMGB-1, High mobility group box-1 protein; HMP, Hypothermic machine perfusion; HOPE, Hypothermic oxygenated perfusion; H&E, Hematoxylin and Eosin; IC, Ischemic cholangiopathy, ICAM-1, Intercellular adhesion molecule-1; IL, Interleukin; IRF3, Interferon regulatory factor 3; IRI, Ischemia-reperfusion injury; IS, Immunosuppression; ITBL, Ischemic type biliary lesions; KC`s, Kupffer cells; LPS, Lipopolysaccharide; LT, Liver Transplantation; MAVS, Mitochondrial antiviral signaling (protein); MELD, Model of end stage liver disease; MPS, Machine perfusion solution; MPT pore, Mitochondria permeability transition pore; mtROS, mitochondrial reactive oxygen species; MyD88, Myeloid differentiation primary response 88; NAD/NADH, nicotine adenine dinucleotide (oxidized/reduced); NFkB, nuclear factor kappa-light-chain-enhancer; NOX4, NMP, Normothermic machine perfusion; NRP, normothermic regional perfusion; PNF, Primary non function; PV, portal vein; RCT, Randomized controlled trial; RET, reverse electron flow; ROS, reactive oxygen species; SCS, standard cold storage; SDH, Succinate dehydrogenase; SEC, sinusoidal endothelial cells; TIRAP, Toll-interleukin 1 receptor (TIR) domain containing adaptor protein; TLR-4 (9), Toll-like-receptor-4; 8-OHdG, 8-hydroxy-2-deoxy Guanosine

## Glossary

**Table:**

ALT	Alanine Aminotransferase
AP-1	Activator Proteine-1
AS	Anastomotic strictures
AST	Aspartate-Aminotransferase
ATP	Adenosine-trisphosphate
BACH2	transcription regulator protein
CD	Cluster of differentiation
CIT	Cold ischemia time
CS	cold storage
CSR	Class switch recombination
Damp`s	Danger associated molecular pattern`s
DBD	donation after brain death
DCD	Donation after circulatory death
DMM	dimethylmalonate
DWIT	donor warm ischemia time
EAD	Early allograft dysfunction
ECD	Extended Criteria Donor
FMN	Flavin-mononucleotide
HA	Hepatic artery
GSH	Glutathione
HAR	hexa-ammineruthenium
HAT	Hepatic artery thrombosis
HMGB-1	High mobility group box-1 protein
HMP	Hypothermic machine perfusion
HOPE	Hypothermic oxygenated perfusion
H&E	Hematoxylin and Eosin
IC	Ischemic cholangiopathy
ICAM-1	Intercellular adhesion molecule-1
IL	Interleukin
IRF3	Interferon regulatory factor 3
IRI	Ischemia-reperfusion injury
IS	Immunosuppression
ITBL	Ischemic type biliary lesions
KC`s	Kupffer cells
LPS	Lipopolysaccharide
LT	Liver Transplantation
MAVS	Mitochondrial antiviral signaling (protein)
MELD	Model of end stage liver disease
MPS	Machine perfusion solution
MPT pore	Mitochondria permeability transition pore
mtROS	mitochondrial reactive oxygen species
MyD88	Myeloid differentiation primary response 88
NAD/NADH	nicotine adenine dinucleotide (oxidized/ reduced)
NFkB	nuclear factor kappa-light-chain-enhancer
NOX4, NMP	Normothermic machine perfusion
NRP	normothermic regional perfusion
PNF	Primary non function
PV	portal vein
RCT	Randomized controlled trial
RET	reverse electron flow
ROS	reactive oxygen species
SCS	standard cold storage
SDH	Succinate dehydrogenase
SEC	sinusoidal endothelial cells
TIRAP	Toll-interleukin 1 receptor (TIR) domain containing adaptor protein
TLR-4 (9)	Toll-like-receptor-4
8-OHdG	8-hydroxy-2-deoxy Guanosine
